# Systemic CD4 Immunity as a Key Contributor to PD-L1/PD-1 Blockade Immunotherapy Efficacy 

**DOI:** 10.3389/fimmu.2020.586907

**Published:** 2020-11-30

**Authors:** Miren Zuazo, Hugo Arasanz, Ana Bocanegra, Gonzalo Fernandez, Luisa Chocarro, Ruth Vera, Grazyna Kochan, David Escors

**Affiliations:** ^1^Oncoimmunology Group, Navarrabiomed, Fundación Miguel Servet-Complejo Hospitalario de Navarra-UPNA-IdISNA, Pamplona, Spain; ^2^Department of Oncology, Complejo Hospitalario de Navarra-IdISNA, Pamplona, Spain

**Keywords:** CD4, immune checkpoint inhibitor, PD-L1, PD-1, biomarker

## Abstract

PD-L1/PD-1 blockade immunotherapy has significantly improved treatment outcome for several cancer types compared to conventional cytotoxic therapies. However, the specific molecular and cellular mechanisms behind its efficacy are currently unclear. There is increasing evidence in murine models and in patients that unveil the key importance of systemic immunity to achieve clinical responses under several types of immunotherapy. Indeed, PD-L1/PD-1 blockade induces the expansion of systemic CD8+ PD-1+ T cell subpopulations which might be responsible for direct anti-tumor responses. However, the role of CD4+ T cells in PD-L1/PD-1 blockade-induced anti-tumor responses has been less documented. In this review we focus on the experimental data supporting the “often suspected” indispensable helper function of CD4 T cells towards CD8 effector anti-tumor responses in cancer; and particularly, we highlight the recently published studies uncovering the key contribution of systemic CD4 T cells to clinical efficacy in PD-L1/PD-1 blockade therapies. We conclude and propose that the presence of specific CD4 T cell memory subsets in peripheral blood before the initiation of treatments is a strong predictor of responses in non-small cell lung cancer patients. Therefore, development of new approaches to improve CD4 responses before PD-L1/PD-1 blockade therapy could be the solution to increase response rates and survival of patients.

## Introduction

Immunotherapies based on PD-L1/PD-1 blockade have revolutionized the treatment paradigm for several cancer types. The PD-L1/PD-1 immune checkpoint inhibitory interaction regulates the activation of immune responses and specifically of T cell responses in physiological conditions. However, cancer cells utilize this strategy to evade from anti-tumor immune responses, but also contributing to a general state of immunosuppression. Many tumor cells upregulate PD-L1, that subsequently binds to PD-1 on the surface of tumor infiltrating T cells (TILs). This interaction inhibits T cell responses by several mechanisms (1, 2). Currently, most PD-L1/PD-1 inhibitors consist of recombinant antibodies that interfere with this T cell inhibitory signal. Therefore, these inhibitors reinvigorate anti-tumor T cell responses and induce the effective elimination of tumor cells. In 2014, pembrolizumab was approved as the first PD-1 inhibitor for the treatment of metastatic melanoma. Since then, the Food and Drug Administration (FDA) has approved nivolumab and pembrolizumab as PD-1 inhibitors, and atezolizumab, durvalumab and avelumab as PD-L1 inhibitors for the treatment of several metastatic cancer types (**Table 1**). These treatments have achieved impressive clinical results characterized by durable responses and prolonged survival of patients compared to conventional therapies. Yet, only a small group of patients obtain clinical benefit while a significant number of patients are still refractory. In addition, there is accumulating evidence supporting that PD-L1/PD-1 blockade in a certain group of patients does accelerate tumor progression and death, an adverse event termed hyperprogressive disease (3). Therefore, there is a critical need for the identification of accurate predictive biomarkers to discriminate patients who will potentially benefit from PD-L1/PD-1 blockade immunotherapies from those that will not respond or can develop hyperprogression. Moreover, the precise molecular mechanisms by which T functions are stimulated by PD-L1/PD-1 inhibitors remain to be fully understood. Such understanding is highly relevant not only for the discovery of predictive biomarkers, but also to designing complementary approaches that may increase clinical efficacy of PD-L1/PD-1 blockade therapy. Despite the general assumption that PD-L1/PD-1 inhibitors reinvigorate pre-existing TIL immunity, recent experimental research supports systemic T cell immunity as a key contributor to PD-L1/PD-1 blockade efficacy (4). These treatments induce broad changes in CD8 immunity which correlate with clinical responses, which could be required for efficacy. However, the longstanding fact that proficient CD8 anti-tumor responses largely depend on CD4 help is often underestimated, or at least overlooked. Here, we discuss the evidence supporting the implication of systemic CD4 immunity in achieving clinical responses under PD-L1/PD-1 blockade therapies, with the proposal that indeed the pre-treatment status of CD4 immunity strongly influences therapy outcomes. We also discuss the promising emerging biomarkers based on quantification of peripheral T cell populations, highlighting CD4 T cell memory subpopulations possibly as a key reliable biomarker for patient selection. Research should go in depth into the molecular mechanisms regulating the interplay between CD4 and CD8 T cell responses under PD-L1/PD-1 blockade immunotherapies. These results will provide new insights on potential molecular targets and alternative strategies to boost CD4 immunity in patients to increase PD-L1/PD-1 blockade efficacy.

**Table 1:** FDA-approved PD-L1/PD-1 inhibitors for cancer treatment.

*Drug name*	*Target*	*Cancer types*
*Pembrolizumab*	PD-1	melanoma, non-small cell lung cancer, head and neck squamous cell cancer, urothelial carcinoma, renal cell carcinoma, classical Hodgkin lymphoma, microsatellite instability-high solid cancer, gastric cancer, cervical cancer, hepatocellular carcinoma, Merkel cell carcinoma, primary mediastinal large B-cell lymphoma
*Nivolumab*	PD-1	metastatic small cell lung cancer, metastatic non-small cell lung cancer, metastatic melanoma, metastatic urothelial carcinoma, metastatic colorectal cancer, hepatocellular carcinoma, advanced renal cell carcinoma, classical Hodgkin lymphoma, metastatic head, and neck squamous cell cancer,
*Atezolizumab*	PD-L1	urothelial carcinoma, non-small cell lung cancer, small cell lung cancer, triple-negative breast cancer
*Durvalumab*	PD-L1	locally advanced non-small cell lung cancer, small cell lung cancer, metastatic urothelial carcinoma
*Avelumab*	PD-L1	locally advanced or metastatic urothelial carcinoma, metastatic Merkel cell carcinoma

## CD4 T Cells as Central Players in Anti-Tumor Immunity

The cancer immunity cycle summarizes how the development of T cell-derived specific anti-tumor responses are essential for controlling tumor growth. Dying cancer cells release tumor-specific antigens (TAA) which are captured by dendritic cells (DC) to prime tumor-specific effector T cells in secondary lymphoid organs. So far, CD8 effector responses have been classically considered as the major players in anti-tumor immunity due to their potent cytotoxicity which enables direct tumor-cell killing (5). Upon antigen recognition, CD8 T cells expand and differentiate into cytotoxic T lymphocytes (CTL) which migrate through peripheral blood and infiltrate tumors. Cancer cells can escape by several mechanisms through a process of immunological editing (6), which includes down-regulation of MHC-I to prevent CD8 T cell recognition (7). In contrast, the importance of CD4 immunity for anti-tumor responses is less recognized as a result of limited studies. Nevertheless, the existing experimental evidence supporting the important role of CD4 T cells is compelling, by promoting and providing help to innate and adaptive anti-tumor immune responses (8). This is reflected by a stronger selective pressure of mutations in MHC-II-restricted neoantigens compared to MHC-I-restricted neoantigens during tumorigenesis, which reinforces the key contribution of CD4 T cells in cancer immunosurveillance (9). The contribution of the different types of effector CD4 T cell subsets is very diverse. Depending on the cytokine milieu during TCR activation, naïve T cells can differentiate into different effector subsets, characterized by different expression of key transcription factors and cytokine profiles that distinctly influence anti-tumor immunity (10).

**Figure 1:**
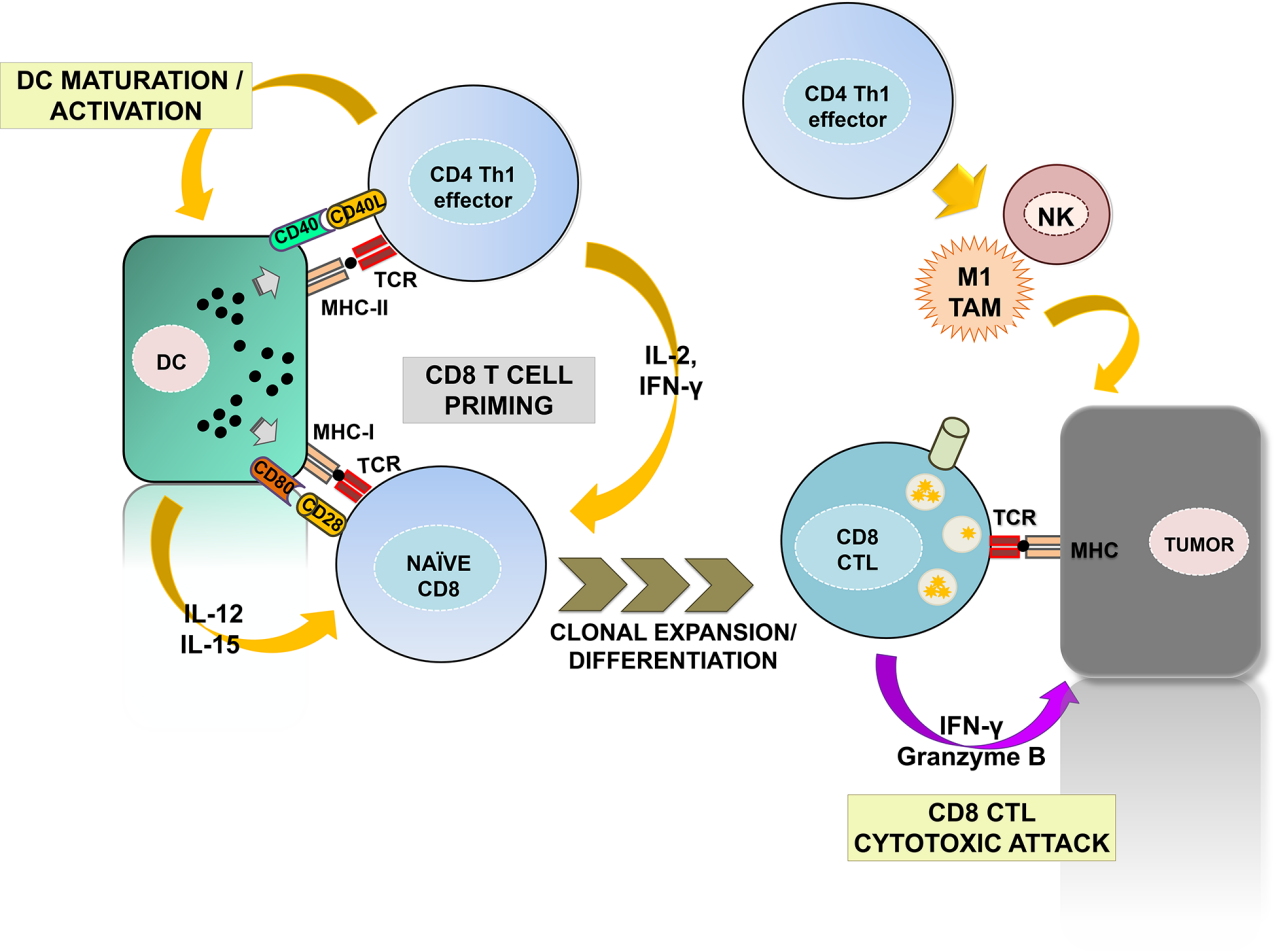
The contribution of CD4 Th1 subsets to anti-tumor immunity. The figure summarizes the well-established roles of CD4 Th1 subsets in anti-tumor responses. Right, CD4 Th1 cells allow the correct priming and differentiation of naïve CD8 T into CTLs by secretion of cytokines and co-stimulatory interactions with DCs within the secondary lymphoid organs. This process termed “DC licensing” leads to DC maturation by CD40L-CD40 binding. CD40-CD40L signaling on DCs induces production of IL-12 and IL-15 and up-regulates co-stimulatory ligands CD80, CD86, and CD70, providing the required signals for CD8 CTL priming. CD80, CD86, and CD70 co-stimulatory ligands on activated DC bind to their receptors CD28 and CD27 on naïve CD8 T cells leading to CTL differentiation and survival. CD8 CTLs infiltrate tumors and exert cytotoxic responses against tumor cells after TAA recognition. Within the tumors, Th1 cells activate NK and M1-macrophages enhancing their innate anti-tumor responses. Th1, T helper 1; CTL, cytotoxic T lymphocyte; DC, dendritic cell; NK, natural killer; M1 TAM, type-1 tumor associated macrophages.

The CD4 helper (Th) 1 subset is the most prominent for anti-tumor immunity (**Figure 1**). Th1 cells promote the priming and differentiation of naïve CD8 T cells into CTLs during antigen presentation by producing cytokines such as IFN-γ and IL-2 (11–13). They also contribute to the maturation and activation of DCs in a process called “DC licensing” through the engagement of CD40L with CD40 on DCs (14–17). Such interaction promotes IL-12 and IL-15 production by DC and up-regulates co-stimulatory ligands CD80 and CD86, providing the required signals for efficient CD8 CTL priming (18, 19). DC activation also favors naïve CD8 T cell recruitment to lymph nodes for priming by yet largely unknown mechanisms (20). Importantly, a recent study has identified the specific gene expression programs by which CD4 Th1 cells provide help for the acquisition of CD8 CTL effector functions, with the CD70-CD27 co-stimulatory pathway playing a prominent role. CD70 up-regulation *via* CD40-CD40L signaling results in co-stimulation of CD8 T cells through binding with CD27, which contributes to CD8 CTL differentiation and clonal expansion (21, 22). In addition, Th1-mediated signaling promotes the establishment of long-lasting CD8 memory (23, 24). Indeed, memory CD8 CTLs primed in absence of CD4 help fail to expand after a second antigen reencounter, and present dysfunctional phenotypes with expression of multiple inhibitory receptors (21, 25, 26). Furthermore, CD4 Th1 cells also activate innate anti-tumor responses by NK and type-1 anti-inflammatory macrophages, promoting tumor cell killing and providing a source of TAAs for T cell priming (27, 28).

Other CD4 T helper subpopulations including Th2 and Th17 have been generally associated with tumor progression. However, several recent studies also show the contrary. CD4 Th2 effector cells could be required for establishing long-term anti-tumor memory responses (29). Likewise, Th17 responses have been reported to induce potent anti-tumor responses in an IFN-γ-dependent manner, and to allow the recruitment of effector cells into the tumor microenvironment (30–34). This “duality of responses” is likely to be context-dependent. Regulatory T cells (Tregs) are key contributors of tolerance by suppressing the other immune cell populations by several means (35–38), such as cell-to-cell contact and production of anti-inflammatory cytokines including IL-10 and TGF-β (39–41). Finally, CD4 T cells can also mediate direct cytotoxic responses through IFN-γ and TNF secretion, production of cytolytic granules or expressing ligand of tumor necrosis factor (TNF) superfamily molecules including FasL or TRAIL leading to cancer cell apoptosis when engaged with their receptors (42–44).

### Differentiation of Memory CD4 T Cells

Upon TAA recognition, CD4 T cells proliferate and differentiate into helper effector T cells. These T cells are short-lived, but a small proportion differentiate into long-lived memory subsets following antigen clearance. Memory T cells undergo fast activation and strong effector responses upon antigen re-encounter (45–47). In humans, the discrimination between the functionally different subsets is based on different expression profiles of cell surface receptors including CD62L and CD45RA. Naïve T cells co-express both CD62L and CD45RA. These T cells exit the thymus and migrate to secondary lymphoid organs driven by CD62L (48). Memory T cells have been divided in two subpopulations based on their location and pattern of migration, either in secondary lymphoid organs (central memory) or in inflamed tissues (effector memory). Central memory T cells express CD62L but not CD45RA, which enable them to circulate between secondary lymphoid organs. In contrast, effector memory T cells are tissue-resident and do not need CD62L nor CD45RA. Effector memory T cells express high levels of chemokine and cytokine receptors to reach inflamed tissues. Finally, the effector population which re-expresses CD45RA (EMRA) is considered a terminally differentiated phenotype which accumulates during lifetime (49). Distinct expression patterns of transcription factors regulate the acquisition of memory phenotypes.

Human CD4 T cells can also be classified in distinct differentiation stages based on CD27/CD28 expression profiles. Following the initial antigen recognition, T cell differentiation advances through the progressive loss of CD27 and CD28 co-stimulatory receptors (50, 51). Hence, human CD4 T cells can be classified into poorly differentiated (CD27^+^ CD28+), intermediately-differentiated (CD27^negative^ CD28+) and highly-differentiated (CD27^negative^ CD28^low/negative^, T_HD_) subsets (51, 52). In humans, T_HD_ cells are largely composed of memory, effector, and senescent T cells.

## CD4 Immunity Has an Important Contribution to PD-L1/PD-1 Blockade Efficacy

The specific molecular mechanisms behind the efficacy of PD-L1/PD-1 blockade therapy have not been fully elucidated yet. These treatments interfere with PD-L1-PD-1 inhibitory interactions by the administration of monoclonal antibodies against both molecules. PD-L1 is overexpressed by several tumor types and confers resistance to pro-apoptotic signals (53, 54); while PD-1 is expressed on T cells upon antigen recognition and interferes with T cell activation when engaged with PD-L1. TILs express high levels of PD-1 and are often functionally imparted due to several dysfunctional states (55). When efficacious, PD-L1/PD-1 blockade therapies seem to counteract tumor-induced T cell dysfunctionality by interfering with PD-1 and PD-L1 signals, and by unleashing activating pathways (1, 56). As a consequence, inhibited tumor reactive T cells reinvigorate and mount an effective anti-tumor response. Indeed, tumor resident CD8+ CD103+ CD69+ T cells which express high levels of PD-1 have been shown to proliferate after PD-L1/PD-1 blockade therapy in melanoma patients (57).

In the last years, increasing evidences have challenged the conventional view of PD-L1/PD-1 inhibitors reinvigorating pre-existing intra-tumoral immunity, supporting that systemic immunity might have a relevant role in therapy efficacy. Various groups have identified that both systemic and intra-tumor CD8 PD-1^+^ T cell subpopulations experience a proliferative burst after PD-L1/PD-1 blockade treatment in several cancer types (57–61). This proliferative burst may be driven by inhibition of the up-regulation of CBL-b ubiquitin ligase by interference with PD-1 signaling in T cells (62, 63). Extensive phenotypic characterization of intra-tumor PD-1+ CD8 T cells shows that this subpopulation is highly heterogeneous, with distinct exhaustion degrees and susceptibility to be reinvigorated by PD-L1/PD-1 blockade (61, 63–66). Interestingly, K.E. Yost and colleagues recently demonstrated that the ability of PD-1 blockade to rescue pre-existing TILs from exhaustion might be limited, demonstrating that expanded TIL clones post-therapy arose from novel clonotypes recruited from the periphery (4). Indeed, two different studies with NSCLC patients have shown that PD-1^+^ CD8 T cells expand systemically following PD-1 blockade therapy and correlated with clinical responses (58, 59). These cells exhibited an effector-like phenotype and expressed co-stimulatory molecules including CD28, CD27, and ICOS. Although it has not been directly demonstrated that this expanded subpopulation was tumor-specific, both studies showed that PD-1 blockade did not alter the proliferation of virus-specific PD-1+ CD8 systemic T cells. The same result was observed in melanoma patients treated with pembrolizumab, with a systemic PD-1+ CD8 subpopulation that expanded after treatment with similar clonotypes to those found in the tumor (60). Additionally, a recent elegant study with melanoma patients confirmed that rearrangements of the peripheral memory cytotoxic CD8 T cell repertoire correlated with response to immune checkpoint blockade (67). As CD8 T cells are possibly the main direct effectors of anti-tumor responses through cytotoxicity over cancer cells, these systemic changes are thought to be the drivers of efficacious clinical responses.

Given the importance of CD4 help function in anti-tumor CD8 responses, it is likely that CD4 responses might be required systemically to achieve efficacious CD8 responses under PD-L1/PD-1 blockade therapy. Pre-clinical studies using murine models and in patients have demonstrated the importance of CD4 immunity for immunotherapy efficacy (68–70). The systemic expansion of a CD4+ CD62L^low^ CD27- FOXP3- CD44+ CXCR3+ ICOS+ T-bet+ T cell subset in mice treated with anti-cancer cell immunoglobulins was correlated with tumor rejection (68). This observation was confirmed in melanoma patients treated with immune checkpoint blockade where a subset of CD4+ PD-1- CD127low T cells was increased in responders compared to non-responders (68). Alspach and colleagues confirmed previously published data on the contribution of CD4 T cells recognizing tumor neoepitopes for the efficacy of immunotherapy (71–73). Moreover, they demonstrated using murine models that both MHC-class I and II-restricted neoantigens are required to generate efficient anti-tumor responses (74). Emerging studies are evaluating the specific contribution of CD4 immunity to PD-L1/PD-1 blockade therapy efficacy which is still unknown. Two recently published studies have independently demonstrated that pre-treatment status of systemic CD4 immunity is a critical factor for determining the clinical outcome of PD-L1/PD-1 blockade therapy in NSCLC patients. Particularly, our study showed that only patients with pre-treatment high numbers of CD4 central and effector memory T cells with a highly differentiated phenotype (CD27-CD28^low/-^) responded to the treatment (75). Responder patients presented high proportion of CD4 memory T cells before treatment initiation. These CD4 T cells exhibited significant proliferative capacities and low co-expression of PD-1/LAG-3 at baseline, and were responsive to PD-1 blockade *ex vivo* and *in vivo* (75). In contrast, patients with low numbers of memory CD4 T cells exhibited a strong CD4 T cell dysfunctionality. Such dysfunctionality was reflected as strongly impaired proliferation capacities and high co‐expression of LAG‐3/PD‐1 associated to resistance to PD-L1/PD‐1 blockade *ex vivo* and *in vivo*. Although all patients showed a baseline dysfunctional CD8 proliferative response, this was only recovered by PD-L1/PD-1 blockade in patients with high numbers of CD4 memory T cells harboring functional proliferative CD4 responses before treatment initiation. These results strongly suggest the implication of PD-1/LAG-3 signaling on systemic T cells dysfunctionality which is under investigation in order to provide new molecular targets to restore CD4 pre-treatment immunity before PD-L1/PD-1 blockade treatment application. Kobayashi and colleagues simultaneously obtained similar conclusions by identifying an equivalent CD4+ CD62L^low^ T effector memory population which was present at higher numbers in responders before PD-1 blockade treatment initiation (76). Patients who maintained high numbers of CD62L^low^ CD4 T cells were long survivors while decreased levels after therapy resulted in acquired resistance. Gene expression analysis of the specific subset revealed that they represented TCR-engaged proliferating Th1-like cells. Indeed, these cells expressed genes related to CD4 help functions involved in the promotion of CD8 CTL responses (76). Hence, the maintenance of systemic CD4 responses over time is required for therapy efficacy. Overall, these two studies provide strong evidence that proficient systemic CD4 immunity is a key factor to achieve efficacious clinical responses under PD-L1/PD-1 blockade therapies.

Although CD4 memory help functions seem to directly influence PD-L1/PD-1 inhibitor effects on CD8 anti-tumor responses, the molecular mechanisms by which both populations interplay during PD-L1/PD-1 blockade therapy remain to be fully understood. In addition, it is not clear yet whether CD4 memory T cells could also play a role within the tumor microenvironment under PD-L1/PD-1 blockade therapy. A recent study in Classic Hodgkin lymphoma (CHL) murine models demonstrated that PD-1 blockade therapy has strong anti-tumor effects on MHC-II expressing tumors mediated by cytotoxic CD4+ T cells (77). Moreover, they observed that CHL patients responding to PD-1 blockade therapy exhibited high CD4+ T cell infiltration compared to non-responders (77). Another study using murine models demonstrated that the expansion of tumor infiltrating follicular CD4+ PD1+ T cells after PD-1 blockade therapy correlated with enhanced CD8 CTL anti-tumor responses and tumor growth control (78). They proposed that this specific population might be an important target of PD-1 blockade within the tumor microenvironment. In contrast, a clinical study with NSCLC patients revealed that the accumulation of CD4+ FOXP3- PD-1 high T cells within the tumor and peripheral blood correlated with higher tumor burden. Moreover, the decrease of such population during therapy onset was significantly associated with improved OS (79) suggesting that those cells might be exhausted. Although the data published so far suggests that exhausted T cell populations within the tumor microenvironment might not be the predominant target of PD-L1/PD-1 blockade therapy, further research is needed to reveal the specific contribution of the different intra-tumoral CD4 T cell subsets to PD-L1/PD-1 blockade efficacy in different cancer types.

## CD4 Immunity as a Reliable Biomarker of Response to PD-L1/PD-1 Blockade Therapy

Most of the current research has been focused on the tumor cell and the immunological status of the tumor microenvironment to find biomarkers of response to PD-L1/PD-1 blockade. These biomarkers include tumor PD-L1 expression, mutational and neoantigen burden, TAA-specific repertoire, presence, and characterization of TILs and infiltration with immunosuppressive cells. PD-L1 tumor expression is the only predictive biomarker accepted so far, but its reliability is still under debate. Recently, tumor mutational burden (TMB) quantification and TIL gene expression have demonstrated potential predictive value in patients treated with PD-L1/PD-1 inhibitors (80–83). Nevertheless, the identification of biomarkers using tumor sampling is challenging for most cancer types, particularly in advanced stages due to limited accessibility. Moreover, single tumor samples do not often represent the tumor heterogeneity. It is important to remark that PD-L1/PD-1 inhibitors are administered systemically and will have a direct impact on systemic immunity, which in turn could correlate with clinical responses (58, 59). Hence, characterization of peripheral immune cell populations of patients undergoing PD-L1/PD-1 blockade therapy is a promising non-invasive source of biomarkers of response, more homogeneous and less costly than tumor sampling. Nevertheless, no peripheral biomarkers approved by the FDA, EMA, and PMDA have been validated for clinical application. Here, we highlight the most promising findings on peripheral biomarkers in predicting clinical outcome to PD-L1/PD-1 inhibitors (**Table 2**).

**Table 2:** Proposed biomarkers of clinical response to PD-L1/PD-1 blockade immunotherapy based on peripheral immune cell populations.

ICI treatment	N of patients	Cancer type	Method of assessment	Association with clinical outcome	Reference
**Anti-PD-1**	30	Metastatic melanoma	CYTOF	Increased baseline CD14+ CD16- HLA-DR^high^ monocytes correlated with high RR and PFS	Krieg et al. (84)
**Anti-PD-1**	67	Metastatic melanoma	CYTOF	Increased baseline CD69+ MIP-1β+ NK cells in responders	Subrahmanyam et al. (85)
**Anti-PD-L1/PD-1**	64	NSCLC	multiple-parametric FC	High PD-1, PD-L1, and PD-L2 expression on PBMC associated with worse OS	Arrieta and Montes-Servín (86)
**Anti-PD-L1/PD-1**	29	NSCLC	Conventional FC	Early on-treatment proliferative responses in CD8+ PD-1+ T cells associated with response	Kamphorst et al. (59)
**Anti-PD-1**	31 33 46	TET and NSCLC	multiple-parametric FC	CD8+ PD-1+ Ki-67_D7/D0_ ≥2.8 associated with better response	Kim et al. (58)
**Anti-PD-1**	29	Metastatic melanoma	High dimensional FC	Ratio of Tex-cell reinvigoration to tumor burden associated with clinical outcome	Huang et al. (60)
**Anti-PD-1**	54	Metastatic melanoma	High dimensional FC	Expansion of CD8+ CCR7- CD27- memory effector T cells correlated with response	Valpione et al. (67)
**Anti-PD-1**	40 43	NSCLC Metastatic melanoma	multiple-parametric FC	Baseline high T cell (CD4 and CD8) CM/effector ratio associated with longer PFS	Manjarrez-Orduño et al. (87)
**Anti-PD-L1/PD-1**	51	NSCLC	Conventional FC	Baseline CD4 T_HD_ (CD27- CD28low/-) >40% associated with response and PD-L1 positivity	Zuazo et al. (75)
**Anti-PD-1**	40 86	NSCLC	multiple-parametric FC	Formula with the ratio between CD4+ CD62L^low^ and CD4+ FOXP3+ CD25+ > 192 associated with response	Kagamu et al. (76)

NSCLC, Non-small-cell lung carcinoma; TET, Thyroid epithelial tumors; RR, Response rate; PFS, Progression free survival; CYTOF, single cell mass cytometry; FC, flow cytometry; Tex, exhausted T cells; CM, central memory; NK, Natural killer; T_HD_; highly differentiated T cells.

A study evaluating peripheral blood samples of NSCLC patients treated with anti-PD-1 by conventional flow cytometry demonstrated that early expansion of peripheral PD-1+ CD8 T cells was associated with clinical efficacy (59). Another study in patients with thymic epithelial tumors confirmed the previous observation (58). The authors correlated peripheral expansion of PD-1+ CD8 T cells with durable clinical benefit to anti-PD-1 treatment. In this case, T cell expansion was measured as the fold-change in the percentage of Ki67+ cells (Ki-67_D7/D0_) ≥2.8 after the first week of treatment. The predictive value of Ki-67_D7/D0_ ≥2.8 was validated in two independent cohorts of NSCLC patients treated with PD-1 inhibitors (58). Early clonal expansion of peripheral T populations evaluated by genome-wide sequencing was also positively associated with clinical responses in NSCLC patients under PD-1 blockade therapy (88). Studies with metastatic melanoma patients treated with PD-1 inhibitors have confirmed this observation. A high ratio of baseline Ki67+PD1+CD8 T subpopulation to tumor burden was associated with longer PFS (60). A recent study from Richard Marais and colleagues using high dimensional flow cytometry also identified the correlation between the expansion of a systemic subset of CCR7- CD27- CD8 cytotoxic memory effector T cells with response to PD-1 blockade in melanoma patients (67). All these studies support the quantification of proliferating peripheral CD8 PD-1+ CD8 T cell subpopulations as a surrogate biomarker to predict responses to PD-L1/PD-1 blockade therapy. These approaches can help decision-making in the early onset of the treatment, but do not identify deleterious adverse events such as hyperprogressive disease. Hence, the power of CD8 T cell subsets to discriminate clinical responses before PD-L1/PD-1 blockade treatment initiation are so far limited.

Detailed characterization of pre-treatment peripheral immune cell population profiles has been performed to identify potential biomarkers for patient selection before treatment initiation. For example, mass cytometry (CYTOF) allows the identification of multiple markers simultaneously. CYTOF-based analyses of peripheral blood from metastatic melanoma patients before anti-PD-1 treatment initiation have identified several potential biomarkers. Increased baseline peripheral CD14+ CD16- HLA-DR^high^ monocytes correlated with a high response rate and PFS (84). High numbers of CD69+ MIP-1β+ NK cells are found in responders compared to non-responders (85). Analyses of PBMC in NSCLC patients by multi-parametric flow cytometry have identified high PD-1, PD-L1 and PD-L2 expression associated with worse OS (86). Although associations with clinical benefit and survival have been observed, none so far have been validated as predictive biomarkers in prospective studies. Moreover, the high cost of these technologies makes it difficult to standardize and implement them in clinical practice.

Work from our group and from other independent groups is demonstrating the value of baseline CD4 memory T cell quantification to predict the efficacy of PD-L1/PD-1 blockade therapy. A preliminary study analyzing 4 metastatic melanoma patients showed that patients with longer survival had an increase in central memory CD4 T cells (89). Another small-scale study with NSCLC patients treated with nivolumab also uncovered higher ratios between systemic central memory and effector subsets in the total populations of CD4 and CD8 T cells was associated with benefit (87). In the last year, two prospective studies which include our own have independently monitored the dynamics of systemic CD4 T cell populations in NSCLC patients undergoing PD-L1/PD-1 blockade therapy as second line treatments (75, 76). Apart from demonstrating that CD4 T cells play a crucial role in PD-L1/PD-1 blockade efficacy, both studies proposed similar relative percentages of peripheral CD4 memory T cells before the start of immunotherapies with strong predictive capacities for clinical benefit. We identified that relative percentages of systemic CD27- CD28^low/-^ central and effector memory CD4 T cells discriminate NSCLC patients with differential clinical outcome (75). A cut-off value of >40% of this subset at baseline identified a subgroup of patients containing the objective responders with an ORR 50%; while patients with <40% at baseline showed an ORR of 0% and were significantly associated with a higher risk for hyperprogressive disease (75). Independently, K. Kobayashi and colleagues identified a CD4+ CD62L^low^ effector memory subset by multiparametric flow cytometry which also discriminates patients with distinct clinical outcome, and with similar threshold values found by Zuazo and collaborators (76). Responders to PD-1 blockade therapy presented a significantly higher proportion of baseline CD62L^low^ CD4 T cells. In contrast, percentages of Tregs were significantly higher in non-responders. Hence, the authors proposed an algorithm accounting for the ratio between CD62L^low^ and CD25+Foxp3+CD4+ T cell population percentages to discriminate responders with a cut-off value of ≥192. Long-term responders maintained a high number of CD62L^low^ CD4 T cell subsets during therapy. In contrast, decreased CD62L^low^ CD4 T cell percentages were an indicator of acquired resistance. Importantly, the results were validated in an independent NSCLC patient cohort.

Finally, multiple studies have evaluated blood parameters derived from routine clinical analyses for correlations with responses to PD-L1/PD-1 blockade immunotherapy. Some of these include absolute neutrophil counts (ANC), absolute lymphocyte counts (ALC), neutrophil-to-lymphocyte ratio (NLR), absolute eosinophil counts (AEC), absolute monocyte counts (AMC), and serum lactate dehydrogenase (LDH). In a cohort of advanced NSCLC patients treated with nivolumab, a baseline ANC ≥7,500/ul correlated with worse PFS and OS (90). Pre-treatment NLR ratios greater than five were associated with decreased PFS and OS in several studies across different cancer types treated with PD-L1/PD-1 inhibitors (91–93). The derived NLR (dNRL) seems to be more consistent as a biomarker, as this index includes monocytes and other granulocyte populations. High dNRL (> 3) was associated with worse OS in NSCLC patients treated with PD-1 blockers. dNRL greater than 3 together with lactate dehydrogenase (LDH) values greater than the upper limit of normal (ULN) have been integrated into a parameter termed “lung immune prognostic index”. This parameter efficiently identifies 3 groups of patients with good (0 factor), intermediate (1 factor), and poor survival (2 factors) under PD-L1/PD-1 blockade therapy (94). Nevertheless, parameters such as ANC, NLR, dNLR, and AMC do not accurately discriminate the wide ranges of myeloid-derived populations at different activation stages in peripheral blood which may play distinct roles in therapy.

## Discussion and Future Perspectives

Most of the research is centered on the immunological status of the tumor microenvironment as a major driving force for the efficacy of PD-L1/PD-1 blockade. However, it is often ignored the significant impact that these treatments exert over systemic immunity and the diverse susceptibility of TIL populations to be reactivated by them. Indeed, several studies have demonstrated that these therapies cause systemic changes in immune cell populations which can be correlated with clinical efficacy (4, 58, 59). Here we wanted to summarize the most relevant studies in which peripheral blood immune cell populations are analyzed in patients undergoing PD-L1/PD-1 blockade therapy. Many of these studies are difficult to validate and implement in clinical routine due to the complexity and high cost of the employed analytical technology. Moreover, biomarkers based on blood cell parameters such as “lung immune prognostic index” have demonstrated to possess rather a prognostic value, while others cannot be cross-tested due to the lack to discrimination between different immune cell populations. This is especially important in studies with myeloid-derived populations (95).

Several of studies reviewed here have followed the dynamics of CD8 T cells and have demonstrated that changes on systemic PD-1+ CD8 subpopulation post-PD-L1/PD-1 blockade therapy might reflect significant anti-tumor activities in patients (59, 60). Moreover, emerging studies suggest that these changes may depend on systemic CD4 immunity (75, 76). Interestingly, they have also revealed that high systemic numbers of CD4 memory T cells assessed by peripheral blood samples before PD-L1/PD-1 blockade treatment initiation can serve as a reliable predictive biomarker in NSCLC patients. Although more extensive independent validation cohorts for distinct cancer types are required to assure its reliability as a predictive biomarker, the statistically powerful ROC curves in both studies supports its clinical application. Moreover, although the proposed CD4 memory populations are very likely equivalent, each study applied different phenotypic markers for their identification. Unification of data provided by both studies will help to better select the specific CD4 memory subpopulation with the strongest predictive value. In addition, dynamic changes of CD4 T-cell memory populations could be successfully used for “real-time” monitoring of responses from blood samples during immunotherapy, to identify early progressors and patients with a high risk of developing hyperprogression (75, 96). These studies together with emerging evidences supporting the limited susceptibility of TILs to be reinvigorated by PD-L1/PD-1 blockade, are indicating that PD-L1/PD-1 inhibitors might be predominantly targeting systemic immunity, which is then recruited into the tumor. The enhancement of systemic CD8 anti-tumor responses by PD-L1/PD-1 inhibitors could possibly be orchestrated by CD4 T cells in the periphery. Hence, these evidences provide the rationale to design new approaches to boost CD4 functionality in cancer patients before their enrolment in PD-L1/PD-1 blockade immunotherapy. Indeed, we showed that increased co-expression of immune-checkpoint molecules PD-1/LAG-3 in systemic T cells confers resistance to PD-1/PD-L1 inhibitors (75). PD-1/LAG-3 co-signaling induce dysfunctional proliferative capacities on systemic CD4 T cells, hampering the help functions over peripheral CD8 responses. The combination of PD-1 and LAG-3 blockade has been shown to reverse CD4 dysfunctionality *ex vivo* (75). In addition, given the contribution of CD40 and CD70 signaling pathways to CD4 helper functions, these molecules represent good molecular targets to reinvigorate CD4 helper functions. Based on data from murine models, the combination of PD-1 inhibitors with CD27 agonistic antibodies seems to be a promising approach to enhance the correct T cell priming and expansion of PD1+ CD8 circulating T cells (97). Cytokine-based therapy with IL-12 has also demonstrated to reinvigorate CD8 expansion during PD-1 blockade *ex vivo* (unpublished observation). Hence, deciphering the molecular mechanisms regulating CD4 helper functions under PD-L1/PD-1 blockade therapy will provide insights into new potential targets, and better approaches to increase PD-L1/PD-1 blockade therapy efficacy.

## Author Contributions

The first version was drafted by MZ and HA. All authors contributed to the article and approved the submitted version.

## Funding

The authors are supported and funded by the Spanish Association against Cancer (AECC, PROYE16001ESCO), Instituto de Salud Carlos III (ISCIII-FEDER, FIS. PI17/02119), and a Biomedicine Project grant from the Department of Health of the Government of Navarre (BMED 050-2019).

## Conflict of Interest

The authors declare that the research was conducted in the absence of any commercial or financial relationships that could be construed as a potential conflict of interest.
